# Genetic and metabolic engineering challenges of C1-gas fermenting acetogenic chassis organisms

**DOI:** 10.1093/femsre/fuab008

**Published:** 2021-02-17

**Authors:** Barbara Bourgade, Nigel P Minton, M Ahsanul Islam

**Affiliations:** Department of Chemical Engineering, Loughborough University, Loughborough, Leicestershire, LE11 3TU, UK; BBSRC/EPSRC Synthetic Biology Research Centre (SBRC), School of Life Sciences, University Park, University of Nottingham, Nottingham, Nottinghamshire, NG7 2RD, UK; Department of Chemical Engineering, Loughborough University, Loughborough, Leicestershire, LE11 3TU, UK

**Keywords:** acetogen, gas fermentation, genetic engineering, metabolic engineering, biotechnology, fuels and chemicals

## Abstract

Unabated mining and utilisation of petroleum and petroleum resources and their conversion to essential fuels and chemicals have drastic environmental consequences, contributing to global warming and climate change. In addition, fossil fuels are finite resources, with a fast-approaching shortage. Accordingly, research efforts are increasingly focusing on developing sustainable alternatives for chemicals and fuels production. In this context, bioprocesses, relying on microorganisms, have gained particular interest. For example, acetogens use the Wood-Ljungdahl pathway to grow on single carbon C1-gases (CO_2_ and CO) as their sole carbon source and produce valuable products such as acetate or ethanol. These autotrophs can, therefore, be exploited for large-scale fermentation processes to produce industrially relevant chemicals from abundant greenhouse gases. In addition, genetic tools have recently been developed to improve these chassis organisms through synthetic biology approaches. This review will focus on the challenges of genetically and metabolically modifying acetogens. It will first discuss the physical and biochemical obstacles complicating successful DNA transfer in these organisms. Current genetic tools developed for several acetogens, crucial for strain engineering to consolidate and expand their catalogue of products, will then be described. Recent tool applications for metabolic engineering purposes to allow redirection of metabolic fluxes or production of non-native compounds will lastly be covered.

## Teaser

This review systematically discusses the challenges of genetically modifying acetogenic chassis, and the recent development of several genetic tools applied to engineer these industrially important microbes for sustainable production of fuels and chemicals from greenhouse gases using C1-gas fermentation.

## INTRODUCTION

The modern economy and industry still rely almost entirely on fossil fuel resources for energy, chemicals, and fuels. Imminent shortage of these finite resources and alarming environmental carbon footprint, mostly through fossil fuel-based greenhouse gas (GHG) emissions have recently led to a renewed interest in developing sustainable processes to replace our reliance on fossil fuels. In this context, biological processes, mainly microbial fermentation, have gained interest as they allow efficient conversion of carbonaceous substrates into target products. Biofuels from biomass, such as ethanol production by bacteria and yeasts (Soleimani, Adiguzel and Nadaroglu 2017; Tian *et al*. 2017), or acetone, butanol, and ethanol fermentation by Clostridia (Lütke-Eversloh and Bahl 2011; Birgen *et al*. 2019), have historically been the predominant bioprocesses, but they cannot currently compete with fossil fuels volume-wise for use as transportation fuels. In addition, upstream lignin degradation for efficient downstream biofuel production remains challenging and expensive (Geddes, Nieves and Ingram 2011; Xu *et al*. 2018). Therefore, microbial hosts able to utilise alternative substrates, such as single carbon (C1) gases CO and CO_2_, are crucial to overcome these challenges. Acetogens can grow autotrophically on CO_2_ or CO as their sole source of carbon, but also show a great metabolic flexibility through their ability to utilise a wide range of substrates, including methanol, formate or glycolate (Drake *et al*. 1997; Drake, Gößner and Daniel 2008; Müller 2019). They possess the Wood-Ljungdahl pathway (WLP) of carbon fixation (Wood 1952; Drake 1994), which allows the conversion of C1-gases into the biomass precursor acetyl-CoA, acetate, and other species-specific products, such as ethanol or butanol, while generating ATP for growth (Ragsdale, 2004, 2008). Although scaling up can be challenging, gas fermentation is industrially promising and viable as the supply of C1-gases is virtually infinite. In fact, several gas fermentation plants are currently in operation with gas supplies derived from various industries such as steel mills (Liew *et al*. 2016; Köpke and Simpson 2020). Additionally, recent progresses in genetic (Köpke *et al*. 2010; Kita *et al*. 2013; Mock *et al*. 2015; Hoffmeister *et al*. 2016; Basen *et al*. 2018; Cheng *et al*. 2019; Shin *et al*. 2019) and metabolic engineering of acetogens can theoretically allow the expansion of the range of compounds produced by these bacteria to virtually any desired target. Such advances enable not only insertion and expression of heterologous genes required for the synthesis of the chosen target compound, but also improved performance of the obtained strain to manipulate metabolic fluxes and increase product titres.

Acetogenic metabolism and the associated complex energy requirements are now reasonably well understood (Schuchmann and Müller 2014, 2016). The thermophilic acetogen, *Moorella thermoacetica* (Fontaine *et al*. 1942; Collins *et al*. 1994) served as the model organism to describe the WLP and the relevant enzymology over 10 years ago (Drake, Gößner and Daniel 2008; Ragsdale 2008), while more recent studies have further strengthened our knowledge of acetogenic physiology and metabolism (Valgepea *et al*. 2017a,b; Souza *et al*. 2019). In addition, genome sequences (Pierce *et al*. 2008; Humphreys *et al*. 2015; Li *et al*. 2015) and in some cases, genome-scale metabolic models (Nagarajan *et al*. 2013; Islam *et al*. 2015; Norman *et al*. 2019) are available for several acetogens, further supporting the development of genetic tools. Recent research efforts have also consolidated the availability of genetic tools for these host organisms to support rigorous metabolic engineering efforts. An improved genetic toolkit has been developed in the past few years for some mesophilic acetogens, including *Clostridium ljungdahlii* (Tanner, Miller and Yang 1993), *Clostridium autoethanogenum* (Abrini, Naveau and Nyns 1994), *Acetobacterium woodii* (Balch *et al*. 1977), and *Eubacterium limosum* (Roh *et al*. 2011; Kelly *et al*. 2016). Different genetic tools such as inducible promoters (Banerjee *et al*. 2014; Nagaraju *et al*. 2016) and CRISPR (Clustered Regularly Interspaced Short Palindromic Repeats)-Cas tools (Huang *et al*. 2016; Nagaraju *et al*. 2016; Woolston *et al*. 2018; Shin *et al*. 2019) have been adapted for these host organisms, and exploited to improve strain performance through metabolic engineering, as well as to diversify and enhance their metabolic capabilities. While these technological advances greatly strengthen the potential of gas fermentation for commercial implementation, some acetogens with promising industrial value such as the thermophile, *M. thermoacetica* or the butanol-producing acetogen, *Clostridium carboxidivorans* (Liou *et al*. 2005) still present challenges with respect to genetic modification. Although some rudimentary progress has been reported for these two acetogens (Kita *et al*. 2013; Cheng *et al*. 2019), efficient genetic manipulation remains limited as the required genetic tools are lacking. Nonetheless, *M. thermoacetica* has some attractive properties for industrial applications, as its thermophilic properties would reduce gas cooling and contamination risks in bioreactors. The mesophilic acetogen, *C. carboxidivorans* differs from other acetogens in its native capacity to produce butanol. As thermophilic properties are advantageous in an industrial context, another acetogenic thermophile, *Thermoanaerobacterium kivui* (Leigh, Mayer and Wolfe 1981) has also recently attracted interest, leading to the development of genetic tools (Basen *et al*. 2018; Jain *et al*. 2020).

Disparity in the availability of genetic tools prevents equal opportunities for improving the industrial potential of different acetogens. To date, *C. autoethanogenum*, *C. ljungdahlii*, and *A. woodii* stand out as the most genetically accessible acetogens and therefore, the most promising hosts for industrial gas fermentation applications. Other acetogens such as *Clostridium ragsdalei* (Kundiyana *et al*. 2011) or *Clostridium coskatii* (Zahn and Saxena 2012) remain largely understudied. This review will explore the optimised genetic tools currently available for some acetogens and the strategies designed to surmount relevant obstacles. A parallel comparison will also be drawn between the progress made and the challenges still faced for other acetogens, for which previously described strategies might be applicable. As for many non-model organisms, successful introduction of foreign DNAs in acetogens depends on overcoming several barriers, including plasmid maintenance through plasmid replication and protection against host restriction-modification systems (Yan and Fong 2017). Methods to address these obstacles, described in this review, are crucial to the development of reliable genetic tools. As these tools allow rapid and reliable genetic modifications in hosts, they can further be applied for metabolic engineering purposes, including the production of non-native compounds or the manipulation of metabolic fluxes. Successful metabolic engineering efforts in acetogens will first be briefly summarised in this review followed by additional approaches relevant to achieving various metabolic engineering aims. As metabolic engineering is a broad field and its application in acetogens is rather scarce, only relevant metabolic engineering approaches and their recent or potential implementation in acetogens for further strain engineering purposes will be presented here.

## GENETIC ENGINEERING CHALLENGES TO MODIFY ACETOGENS

### Overcoming physical and biochemical barriers

**Figure 1.**
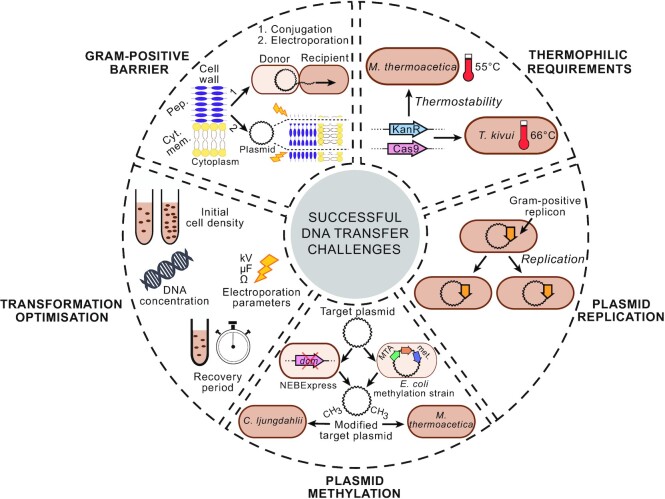
Key challenges associated with a successful DNA transfer into acetogenic hosts. Target vectors must first be inserted into the cells via electroporation or conjugation (Pep: peptidoglycan; cyt. mem.: cytoplasmic membrane). In addition, foreign genes intended for expression into thermophilic acetogens must retain their activity at high temperatures, further complicated exogenous gene expression. To maintain a target plasmid within the bacterial population, a compatible gram-positive replicon is required to add to the plasmid, allowing stable plasmid replication. In addition, transformation plasmids might require pre-methylation prior to transformation into the host to protect them again the host's RM systems (dcm: E. coli dcm methyltransferase; MTA met.: M. thermoacetica native methyltransferases). Different methods have been described for achieving such pre-methylation of plasmids. Lastly, the transformation protocol requires to be optimised to increase the transformation efficiency, i.e., the number of positive transformants/mutants obtained.

The ability of acetogens to convert C1-gases (e.g., CO_2_ and CO) into different products and to use a range of substrates, including hexose sugars and methanol (Drake, Gößner and Daniel 2008; Ragsdale 2008), promote them as valuable chassis organisms for industrial bioprocesses. In addition, strain engineering, mediated by different genetic engineering techniques, can now improve the metabolic performance of acetogens to meet industrial demands. However, robust genetic tools must be in place not only to diversify their applications but also to promote their wider use in the industry. Several challenges, including a reliable DNA transfer method and the lack of an efficient genetic system, have complicated the genetic modification of acetogens. While a few acetogens, such as *C. ljungdahlii* and *C. autoethanogenum*, are now amenable to genetic modifications, DNA transfer in most acetogens is limited by physical and biochemical barriers, including their Gram-positive cell wall or thermophilic growth requirements (Fig. 1). In addition, stable plasmid replication, mediated by a compatible Gram-positive replicon for each acetogen and evasion of native restriction-modification systems to prevent plasmid degradation also stand out as other key obstacles to improved DNA transfer in acetogens (Fig. 1). Once these obstacles are overcome, different genetic tools for strain manipulation can be adapted to create a powerful genetic toolkit for these organisms. Some of these tools, as described below, have already been developed and implemented for several acetogens. It is likely that they can be adapted to make genetic manipulation accessible for most, if not all, acetogens in the near future.

#### DNA transfer into an acetogenic host

First and foremost, an efficient DNA transfer method to introduce and express foreign DNA molecules must be in place to genetically engineer acetogens. Similar to many other Firmicutes, acetogens have a Gram-positive cell wall structure (Fontaine *et al*. 1942; Tanner, Miller and Yang 1993; Abrini, Naveau and Nyns 1994) with a thick layer of peptidoglycan (Fig. 1), therefore harder to disrupt when inserting foreign DNA. Electroporation, which relies on an electric shock to create pores in the membrane, has proven to be the most effective technique to transform many Gram-positive species, including the acetogens *C. ljungdahlii* (Köpke *et al*. 2010; Leang *et al*. 2013), *A. woodii* (Stratz *et al*. 1994), *M. thermoacetica* (Kita *et al*. 2013), and *E. limosum* (Shin *et al*. 2019). Although the protocol employed requires species-specific optimisation, electroporation represents a rapid and easy transformation method for most acetogens. Conjugation has also been employed for DNA transfer in several acetogens such as *C. autoethanogenum* (Mock *et al*. 2015; Nagaraju *et al*. 2016) and *C. carboxidivorans* (Cheng *et al*. 2019). This technique relies on cell-to-cell contact between the donor strain, usually *Escherichia coli* and the receiving host. Although this method is more time-consuming than electroporation, it has been hypothesised to allow partial evasion of the host restriction-modification barriers (Jennert *et al*. 2000; Purdy *et al*. 2002; Cheng *et al*. 2019), as DNA is transferred from the donor to the host strain as a single-stranded molecule during conjugation. In rare cases, hosts might naturally take up the foreign DNA. For example, the thermophilic acetogen *T. kivui* is naturally competent (Basen *et al*. 2018), therefore rendering its transformation straightforward.

#### Thermostability of exogenous enzymes

A limited number of acetogens, notably *M. thermoacetica* and *T. kivui*, are thermophilic organisms, with an optimal growth temperature of 55°C (Fontaine *et al*. 1942) and 66°C (Leigh, Mayer and Wolfe 1981), respectively. Although this thermophilic requirement has several advantages in an industrial context, it complicates their genetic manipulation because thermostability of introduced genetic elements has to be considered. For example, high growth temperatures limit the availability of selection markers as most markers rely on antibiotic resistance. The chosen antibiotic must remain stable at high temperatures for long incubation periods. In addition, the gene product encoded by the antibiotic resistance gene must be functional at the optimal growth temperature. Thermostable versions of several enzymes responsible for antibiotic resistance have already been created, allowing transformant selection in thermophilic hosts. Kanamycin antibiotic, for instance, has been used in several thermophilic anaerobes, including *M. thermoacetica* (Iwasaki *et al*. 2013) and *T. kivui* (Basen *et al*. 2018), using a thermostable kanamycin resistance cassette derived from *Enterococcus faecalis* (Trieu-cuot and Courvalin 1983; Mai, Lorenz and Wiegel 1997). Other antibiotics, such as spectinomycin (Zhou, Wu and Rao 2016), bleomycin (Brouns *et al*. 2005), and hygromycin (Nakamura *et al*. 2005) have also been shown to be functional at high temperatures, but thermostable selection markers remain scarce. As commonly carried out for new potential hosts, minimal inhibitory concentration (MIC) assays (Yan and Fong 2017) can be performed to test the natural antibiotic resistance, antibiotic thermostability, and the required antibiotic concentration to identify the most suitable selection markers for a specific host. Moreover, other products encoded by exogenous genes introduced in these thermophilic acetogens must also meet this thermostable requirement. For example, if a CRISPR-Cas9 tool is intended for use in these organisms, a thermostable version of Cas9, already developed and used in other thermophilic hosts (Mougiakos *et al*. 2017), will be required for the tool to retain its activity. Indeed, as temperature impacts protein folding (Feller 2018), mesophilic proteins might not fold properly at higher temperatures, leading to protein misfunction. To overcome this issue, genes can be engineered to increase thermostability when necessary, as demonstrated for antibiotic resistance genes (Lipscomb *et al*. 2016) or *cas9* (Mougiakos *et al*. 2017). In addition, genes from other thermophiles such as *Clostridium thermocellum* (Groom *et al*. 2016) or *Geobacillus thermoglucosidasius* (Sheng *et al*. 2017) can act as a pool of potential thermostable candidates. Thus, although thermophilic growth is advantageous for industrial applications, relevant enzyme and compound thermostability must be taken into account when designing manipulation techniques.

#### Gram-positive replicon-mediated plasmid replication

Different methods to insert foreign DNA for engineering a host's genome, further explored later in this review, have been reported for acetogens. Indeed, homologous recombination (HR) can be harnessed to insert a specific cassette in the genome, creating a stable mutant strain. The engineering cassette can be harboured on a suicide vector, unable to replicate within the host's cell, or on a replicating plasmid, maintained within the bacterial population. Although mutant strains have successfully been obtained with suicide vectors for some acetogens (Kita *et al*. 2013; Basen *et al*. 2018), this type of vectors tend to be favoured for organisms with a high transformation efficiency, as the cells do not maintain the vector. Replicating plasmids might be a more suitable option for organisms that are harder to transform like acetogens. In addition, some applications such as CRISPR-Cas tools require plasmid-borne expression of different elements, inaccessible without a replicating plasmid. Therefore, engineering a stable shuttle vector, although not a necessity for some applications, stands out as a key step to expand the genetic toolkit available for acetogens. Stable maintenance of a plasmid in a bacterial population requires plasmid replication and partition to each daughter cell during cell division. Accordingly, the replicon, *i.e*., the plasmid module consisting of the origin of replication and replication related genes of an introduced plasmid must be functional in the chosen host organism for stable plasmid maintenance. To date, a range of Gram-positive replicons have been used for successful plasmid replication in several acetogens (Table 1). Four different replicons are, for example, available in the pMTL80000 shuttle vector series (Heap *et al*. 2009), originally created to target clostridial species, and have shown to be equally functional in several acetogens. Indeed, the replicons pBP1 from *Clostridium botulinum*, pCB102 from *Clostridium butyricum*, and pCD6 from *Clostridioides difficile* (formerly *Clostridium difficile*) have enabled successful plasmid maintenance in different acetogens, including *C. autoethanogenum* (Nagaraju *et al*. 2016; Liew *et al*. 2017; Annan *et al*. 2019), *C. ljungdahlii* (Ueki *et al*. 2014; Molitor *et al*. 2016; Woolston *et al*. 2018), and *A. woodii* (Hoffmeister *et al*. 2016; Beck *et al*. 2020). In addition, *A. woodii* (Hoffmeister *et al*. 2016) and *C. ljungdahlii* (Woolston *et al*. 2018) have also been transformed with plasmids harbouring the *Clostridium perfringens* pIP404 replicon (Bannam and Rood 1993). Having multiple Gram-positive replicons available for one species is ideal, as it further diversifies the applicable genetic engineering strategies in relevant hosts. For example, two plasmids with different compatible replicons can replicate simultaneously in a host, increasing the size and number of exogenous genes expressed at once. Annan *et al*. (2019) maintained two plasmids with the pBP1 and pCB102 replicons, respectively, in *C. autoethanogenum* to create a prototroph strain for pantothenate and biotin. As the biosynthetic pathways for pantothenate and biotin were quite large, cloning each of the pathways on a different vector overcame the issue of plasmid size limitation and enabled expression of both pathways simultaneously. A similar two-plasmid system has previously been reported for other applications such as CRISPR-based tools in clostridial species to keep plasmid size relatively small (Wasels *et al*. 2017). These strategies require the availability of at least two compatible Gram-positive replicons, capable of replicating in the presence of each other. The two plasmids should also not share any region of DNA homology to prevent any undesirable recombination events. Additionally, each Gram-positive replicon replicates at a different level in the same host, which impacts plasmid copy numbers and can alter target production. For instance, it was previously reported that four replicons, pIP404, pBP1, pCB102, and pCD6, were all active in *A. woodii* (Hoffmeister *et al*. 2016), but less plasmid copies were maintained in the cells when pCB102 was used. Indeed, acetone production was decreased when an acetone-producing pathway was carried on a plasmid with pCB102 as compared to the three other replicons, leading the authors to hypothesise that the pCB102 replicon maintains fewer plasmid copy numbers in *A. woodii*. Low copy plasmids might be required to reduce metabolic impacts from the expression of toxic genes. Although multiple replicons should be available for a host organism, in some cases, only one compatible replicon can be identified. For example, transformation of *C. carboxidivorans* has only been reported with the pBP1 replicon (Cheng *et al*. 2019). Similarly, the four pMTL80000 replicons and pIP404 were tested in *E. limosum* (Shin *et al*. 2019), but only pIP404 led to the sufficient transformation efficiency. The transformation efficiency obtained with other replicons was extremely low, ranging from 0–1.8 transformants/µg of DNA.

**Table 1.** Gram-positive replicons allowing successful plasmid replication in several acetogens.

Replicon Modified From	Applied Chassis	Reference
*C. perfringens* pIP404^a^	*C. ljungdahlii* *A. woodii* *E. limosum*	Woolston *et al*. 2018 Hoffmeister *et al*. 2016 Shin *et al*. 2019
*C. botulinum* pBP1^b^	*C. autoethanogenum* *C. ljungdahlii* *A. woodii* *C. carboxidivorans*	Annan *et al*. 2019 Ueki *et al*. 2014; Molitor *et al*. 2016 Hoffmeister *et al*. 2016 Cheng *et al*. 2019
*C. butyricum* pCB102^b^	*C. autoethanogenum* *C. ljungdahlii* *A. woodii*	Annan *et al*. 2019; Liew *et al*. 2017; Nagaruju *et al*. 2016 Molitor *et al*. 2016 Hoffmeister *et al*. 2016; Beck *et al*. 2020
*C. difficile* pCD6^b^	*C. autoethanogenum* *A. woodii*	Annan *et al*. 2019 Hoffmeister *et al*. 2016
*T. saccharolyticum* pMU131^c^	*T. kivui*	Basen *et al*. 2018
Suicide vector	*T. kivui* *M. thermoacetica*	Basen *et al*. 2018 Kita *et al*. 2013

^a^Bannam and Rood 1993

^b^From the pMTL80000 series - Heap *et al*. 2009

^c^Shaw, Hogsett and Lynd 2010

While the five discussed replicons have enabled significant progress in transforming several mesophilic acetogens, transformation of thermophilic acetogens presents additional challenges. The naturally competent thermophile, *T. kivui* was successfully transformed with plasmids harbouring the pMU131 replicon from the closely related species *Thermoanaerobacterium saccharolyticum* (Shaw, Hogsett and Lynd 2010) and suicide vectors, *i.e.*, non-replicating plasmids (Basen *et al*. 2018). The authors used *T. kivui*’s natural competence to create a Δ*pyrE* strain with a suicide vector (Basen *et al*. 2018). This non-replicating vector carried a homologous recombination (HR) cassette to delete *pyrE*, a gene coding for an orotate phosphoribosyltransferase that is essential for uracil biosynthesis (Jund and Lacroute 1970). Orotate phosphoribosyltransferase also converts 5-fluoorotic acid (5-FOA) into the toxic compound 5-fluorouracil, allowing counter-selection, as a Δ*pyrE* mutant cannot grow without uracil supplementation but is resistant to 5-FOA. The resulting mutant strain can then be further used for genetic manipulation purposes by re-inserting *pyrE* alongside the desired genetic modification at the target locus, restoring the wild-type uracil phenotype. As this method can be impacted by transformation efficiency and HR frequency, it is more suited to naturally competent *T. kivui*. Similarly, deletion of *pyrF*, encoding an orotidine 5’-phosphate decarboxylase and also essential for the uracil pathway to metabolise 5-FOA, was exploited for genetic manipulation in *M. thermoacetica* (Kita *et al*. 2013). However, since *M. thermoacetica* is not naturally competent, this method was suboptimal in this host, as only one transformant out of 100 colonies screened harboured the desired *pyrF* deletion. Unfortunately, a functional Gram-positive replicon for *M. thermoacetica* has yet to be identified, complicating the development of genetic tools for this acetogen. Moreover, as *pyrF*-mediated genetic manipulation involves cassette integration into the genome (Kita *et al*. 2013; Iwasaki *et al*. 2017), this method may not be adequate for some applications such as CRISPR-Cas tools, which require plasmids to carry essential genetic elements for the tool and replicate within the population. Since no straight-forward method is available to identify compatible replicons for a chosen host, testing other Gram-positive replicons from closely related thermophilic organisms in *M. thermoacetica* is required to achieve successful plasmid replication in this host. Plasmid replication, mediated by a Gram-positive replicon, is essential to maintain shuttle plasmids within the population, a key step for most genetic applications.

#### Native restriction-modification barriers

Acetogens, similar to many other bacterial species, carry native restriction-modification (RM) systems. These systems use methylation patterns to recognise and degrade foreign DNAs, further challenging plasmid introduction into a host. To date, four types of RM systems have been identified in bacteria, with type II being the most common (Roberts *et al*. 2003; Pingoud *et al*. 2005; Vasu and Nagaraja 2013). While types I, II, and III target unmethylated DNA molecules, type IV recognises DNA with foreign methylation patterns. RM systems include restriction endonucleases, which cleave foreign DNAs at specific sequences, and methyltransferases, which methylate the host's genome to protect it from the endonucleases (Vasu and Nagaraja 2013). To circumvent this protective system during the transfer of foreign DNAs into the host, plasmids can be methylated prior to transfer to prevent plasmid recognition and cleavage by the host's restriction system(s) (Fig. 1). To do so, the plasmid is first introduced in a methylation host, invariably *E. coli*, for plasmid methylation. Several strategies for plasmid pre-methylation have previously been described, but the best method will largely be species-specific. In some cases, a commercially available *E. coli* strain, with a different Dam/Dcm background, is sufficient to protect the plasmid. *E. coli* Dam and Dcm methyltransferases methylate adenine and cytosine, respectively (Marinus and Løbner-Olesen 2014). It was previously shown that plasmid methylation by these two enzymes during the cloning steps induced recognition and degradation of the plasmid DNA in some hosts (Kolek *et al*. 2016). To prevent this, plasmids can first be expressed in a *E. coli* mutant strain with a modified Dam/Dcm background to avoid plasmid methylation. It has, for example, been shown that plasmid DNAs isolated from NEBExpress and lacking Dcm (New England Biolabs) yield better transformation results in *C. ljungdahlii* (Leang *et al*. 2013). In fact, this method even offers better transformation efficiency than *in vivo* methylation, and subsequent *C. ljungdahlii* transformations have been performed with plasmids propagated in NEBExpress (Banerjee *et al*. 2014; Woolston *et al*. 2018). This strain, however, cannot be used as a donor in conjugative plasmid transfer, as it lacks the necessary conjugative plasmid machinery. Accordingly, Woods and co-workers constructed a derivative strain, *E. coli* sExpress by transferring into NEBExpress the R-factor R702 from the commonly used *E. coli* conjugative donor strain CA434 (Woods *et al*. 2019). Strain sExpress was shown to act as a more effective conjugative donor with several different clostridial recipients that possessed type IV restrictions systems, including *C. autoethanogenum*, where the number of transconjugants obtained was almost 300 times higher than with the donor strain CA434. In other cases, expression of a methyltransferase from another organism allows for adequate protection against the host's RM system for transformation to occur. However, in most complex cases, the host's native methyltransferases must be expressed in the *E. coli* methylation strain, as described for Plasmid Artificial Modification (PAM) strategy (Yasui *et al*. 2009) and also used for *M. thermoacetica* (Kita *et al*. 2013). To do so, the native methyltransferases, identified from the PacBio sequencing and available in the REBASE database (Roberts *et al*. 2015), are first expressed in *E. coli* to pre-methylate plasmids prior to introducing them into the host organism. This method, *i.e*., expressing in *E. coli* three *M. thermoacetica* genes corresponding to type I and type II RM systems, allowed *in vivo* methylation sufficient to protect plasmids from degradation by *M. thermoacetica* (Kita *et al*. 2013). Notably, *C. carboxidivorans* has arguably the most complex RM profile amongst acetogens, harbouring the putative RM systems of each type (Roberts *et al*. 2015). To date, the recognition sequences associated with these different RM systems have not been identified. However, Cheng *et al*. (2019) showed that conjugation, which transfers the transformation plasmid as a single-stranded DNA molecule, allowed the selection of positive transformants in this organism without requiring plasmid pre-methylation. This improvement was applied to generate mutant strains with enhanced ethanol and butanol production.

#### Optimisation of the transformation protocol

The different elements described above are crucial for successful DNA transfer in acetogenic hosts, but the transformation protocol itself should also be optimised to increase the transformation efficiency, i.e., the success of a transformation or conjugation process. It is, however, important to mention that a high transformation efficiency is not a necessity for some genetic applications. For example, isolating one mutant harbouring the required genetic modification might be sufficient for CRISPR-Cas tools. However, false transformants or spontaneous mutants might arise, and many colonies are usually screened to increase the likelihood of identifying correct mutants. In addition, applications such as the generation of mutant libraries require a large number of mutants, which can be achievable through improving the transformation process. Thus, increasing the transformation efficiency, partly through optimisation of the transformation protocol, is important for many genetic applications. However, optimisation of the transformation protocol is an empirical process, which requires optimisation of a multitude of parameters, including cell density, DNA concentration, electroporation parameters, and cell recovery period. When preparing competent cells, the cell density at which cells are harvested can impact the transformation efficiency. The published transformation protocol for *M. thermoacetica* harvested cells when OD_600_ reached 0.1 – 0.2 to prepare cells for transformation (Kita *et al*. 2013). However, higher cell densities for competent cell preparation have been used for other acetogens, including OD_600_ = 0.2 – 0.3 for *C. ljungdahlii* (Leang *et al*. 2013; Woolston *et al*. 2018) and *A. woodii* (Hoffmeister *et al*. 2016), and even OD_600_ = 0.3 – 0.5 for *E. limosum* (Shin *et al*. 2019). In addition, DNA concentration and DNA purity can influence the transformation outcome. Indeed, residual salts in DNA samples can impede electroporation since this method requires an electric shock. DNA concentrations for acetogens commonly range from 1 μg to 5 μg (Kita *et al*. 2013; Hoffmeister *et al*. 2016; Woolston *et al*. 2018; Shin *et al*. 2019). Furthermore, time-consuming optimisation of key electroporation parameters, such as voltage, resistance, or pulse length, which are dependent on cuvette gap width, cell diameter, and temperature, must be carried out to influence the transformation efficiency. *M. thermoacetica*, for instance, has successfully been electroporated at 1.5 kV, 500 Ω, and 50 μF (Kita *et al*. 2013), while different electroporation parameters, 0.625 kV, 600 Ω, and 25 μF, have been used for *C. ljungdahlii* (Woolston *et al*. 2018), further emphasising the requirement of species-specific optimisation of electroporation parameters. Moreover, the recovery period, performed in a liquid medium with no selective pressure applied, allows the cell culture to reach a higher OD and to express the selection marker gene before plating. A recovery period of 9 – 12 hours has been reported for several acetogens (Leang *et al*. 2013; Woolston *et al*. 2018; Shin *et al*. 2019), whereas the published protocol for *M. thermoacetica* reported a 48-hour recovery period before being transferred onto solid medium (Kita *et al*. 2013). Since there is no selective pressure during recovery, longer periods may lead to plasmid loss. Lastly, a key aspect for transformation is the ability to obtain individual colonies on solidified media. Although this has been achieved for most acetogens (Woolston *et al*. 2018; Shin *et al*. 2019), growth of *M. thermoacetica* on plates has not been reported in the literature, suggesting that this organism is unable to adapt to plates. Instead, CO_2_-filled anaerobic Hungate tubes coated with a thin layer of solid medium have been used for *M. thermoacetica* colony formation (Kita *et al*. 2013). In addition, colony formation also depends on appropriate selection, i.e., sufficient expression of the selection marker gene and appropriate concentration of the selection marker.

### Methods for genetic manipulations

**Figure 2.**
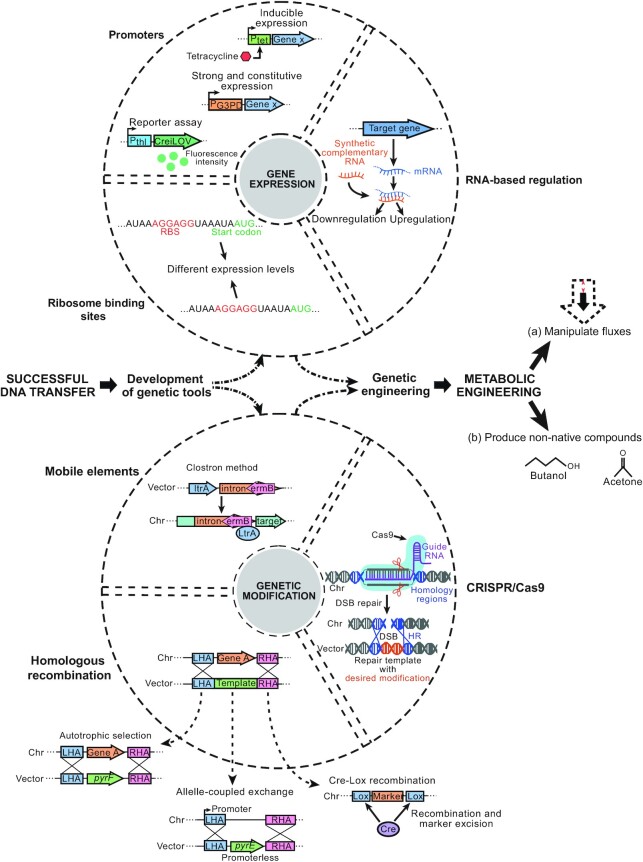
Overview of genetic tools already available for some acetogens. These tools allow to manipulate gene expression levels and to modify the genome. Gene expression can be modified by engineered promoters and ribosome binding sites or be regulated by synthetic RNA molecules. Several methods to modify the host's chromosome have been developed. Most methods rely on homologous recombination for template insertion into the chromosome, and different strategies to select for positive transformants can be applied. Mobile elements, harnessed in the ClosTron method for example, are useful tools to disrupt gene expression, particularly useful to study the host metabolism. Recently developed CRISPR-based methods are powerful tools to impart scarless genetic modifications. These techniques can further be harnessed for metabolic engineering strategies such as manipulation of metabolic fluxes or production of non-native compounds. Chr: chromosome; DSB: double-stranded break; HR: homologous recombination; LHA: left homology arm; RHA: right homology arm; RBS: ribosome binding site.

Once the DNA transfer process has been optimised and a basic shuttle vector is engineered, these methods can be applied for genetic modifications involving the alteration of native gene expression, addition of new attributes, and elimination of native functions through gene knockout or knockdown (Fig. 2). For example, expression of exogenous genes or redirection of metabolic fluxes can be mediated by engineered regulatory elements, including promoters and ribosome binding sites, further described below. Manipulation of gene expression levels is a key aspect of metabolic engineering strategies, as it can control the product yield by the controllable expression of target genes or elimination of competing pathways. Furthermore, other genetic modifications such as mutations or gene deletions can be achieved with several methods, many of which rely on homologous recombination (HR) to insert the plasmid-borne modification template into the host's chromosome. Several HR-based techniques will be described in the following sections, as they have already been successfully applied to some acetogens (Table 2). In addition, the recent CRISPR-Cas tools have simplified genetic engineering efforts by allowing rapid and efficient selection of mutant strains through RNA-guided genome editing. However, adapting such tools to a new host organism can be challenging and, to date, only a few studies have reported the successful use of CRISPR-Cas in acetogens (Huang *et al*. 2016; Nagaraju *et al*. 2016; Woolston *et al*. 2018; Shin *et al*. 2019; Zhao *et al*. 2019).

**Table 2.** Examples of genetic tools adapted for acetogens and their applications for metabolic engineering purposes.

**GENETIC TOOLS**		
**Tool**	**Applied Chassis**	**Reference**
**Promoters: constitutive**		
*M. thermoacetica* G3PD promoter	*M. thermoacetica*	Kita *et al*. 2013
*C. autoethanogenum acsA* promoter, P*_acsA_*	*C. autoethanogenum*	Liew *et al*. 2017
*C. ljungdahlii pta* promoter, P*_pta_*	*C. ljungdahlii; A. woodii*	Ueki *et al*. 2014; Hoffmeister *et al*. 2016
Engineered P4 promoter	*C. ljungdahlii*	Woolston *et al*. 2018
*C. acetobutylicum* thiolase promoter, P*_thl_*	*C. ljungdahlii; A. woodii*	Woolston *et al*. 2018; Hoffmeister *et al*. 2016
*A. woodii ack* promoter	*A. woodii*	Hoffmeister *et al*. 2016
Synthetic *E. coli* Bba_J23119 promoter, P*_thl_*	*E. limosum*	Shin *et al*. 2019
*C. tyrobutyricum* thiolase promoter	*C. carboxidivorans*	Cheng *et al*. 2019
*Thermoanaerobacter* gyrase promoter, P*_gyr_*	*T. kivui*	Basen *et al*. 2018
*T. kivui* S-layer protein promoter	*T. kivui*	Jain *et al*. 2020
**Promoters: inducible**		
Lactose-inducible P*_bgaL_* promoter	*C. ljungdahlii; A. woodii*	Banerjee *et al*. 2014; Woolston *et al*. 2018; Beck *et al*. 2020
Tetracycline-inducible P_2tetO1_ promoter	*C. ljungdahlii*	Woolston *et al*. 2018
Tetracycline-inducible promoter library	*C. autoethanogenum*	Nagaraju *et al*. 2016
Tetracycline-inducible P_tetO1_ promoter	*E. limosum*	Shin *et al*. 2019
Tetracycline-inducible P_tet_ promoter	*A. woodii*	Beck *et al*. 2020
Lactose-inducible P*_fac_* promoter	*A. woodii*	Beck *et al*. 2020
Theophylline-inducible P*_ack-theo_* promoter	*A. woodii*	Beck *et al*. 2020
**Ribosome binding sites**		
RBS modification of the crotonase gene	*C. ljungdahlii*	Ueki *et al*. 2014
RBS optimisation for 3-HB production	*C. ljungdahlii*	Woolston *et al*. 2018
Identification of consensus sequence	*E. limosum*	Song *et al*. 2017
**RNA-based regulation**		
CRISPR interference	*C. ljungdahlii; E. limosum*	Woolston *et al*. 2018; Shin *et al*. 2019
**Homologous recombination**		
*pyrF* selection	*M. thermoacetica;* *C. autoethanogenum*	Kita *et al*. 2013; Liew *et al*. 2017
*pyrE* selection	*T. kivui*	Basen *et al*. 2018
Allele-coupled exchange	*C. ljungdahlii; C. autoethanogenum*	Annan *et al*. 2019
Cre-Lox recombination	*C. ljungdahlii*	Ueki *et al*. 2014
**Mobile elements**		
ClosTron	*C. autoethanogenum; C. ljungdahlii*	Mock *et al*. 2015; Marcellin *et al*. 2016; Bengelsdorf *et al*. 2016
*Hmar1* transposase	*C. ljungdahlii*	Philipps, de Vries and Jennewein 2019
**CRISPR-Cas9**		
CRISPR	*C. autoethanogenum; C. ljungdahlii; E. limosum*	Nagaraju *et al*. 2016; Huang *et al*. 2016; Shin *et al*. 2019
CRISPR interference	*C. ljungdahlii; E. limosum*	Woolston *et al*. 2018; Shin *et al*. 2019
**TOOL APPLICATION**		
**Application**	**Applied Chassis**	**Reference**
**Genetic modification**		
Deletion of *adhE1*	*C. ljungdahlii*	Leang *et al*. 2013; Huang *et al*. 2016; Bengelsdorf *et al*. 2016
Deletion of *ldhA*	*C. autoethanogenum*	Nagaraju *et al*. 2016
Deletion of *adhE*	*C. autoethanogenum*	Liew *et al*. 2017
Deletion of *pduL1/2*	*M. thermoacetica*	Iwasaki *et al*. 2017
Deletion of *fruK*	*T. kivui*	Basen *et al*. 2018
Deletion of HDCR	*T. kivui*	Jain *et al*. 2020
Deletion of the *rnf* operon	*A. woodii*	Westphal *et al*. 2018
Deletion of the *lctBCDEF* operon	*A. woodii*	Schoelmerich *et al*. 2018
Deletion of *hydAB*	*A. woodii*	Weichmann *et al*. 2020
Insertion of an ADI pathway	*A. woodii*	Beck *et al*. 2020
**Production of non-native compounds**		
Butanol	*C. ljungdahlii*	Köpke *et al*. 2010
Butyrate	*C. ljungdahlii*	Ueki *et al*. 2014
Acetone	*C. ljungdahlii; A. woodii*	Banerjee *et al*. 2014; Hoffmeister *et al*. 2016
Ethanol	*M. thermoacetica*	Rahayu *et al*. 2017
Lactate	*M. thermoacetica*	Iwasaki *et al*. 2017
Isoprene and mevalonate	*C. ljungdahlii*	Diner *et al*. 2018

#### Manipulation of gene expression levels

Manipulating gene expression levels (Fig. 2), an important aspect of strain engineering, can be mediated by regulatory elements such as promoters and ribosome binding sites (RBS), as well as other genetic engineering strategies such as RNA-based tools (Chae *et al*. 2017).

##### Promoters

Promoters directly impact gene transcription and therefore, gene expression levels. Identifying compatible promoters for a specific host organism allows expression of introduced exogeneous genes, while promoter engineering can further manipulate expression levels of both native and non-native genes for strain engineering purposes. Despite their importance, promoter activity and strength have not been extensively studied in most acetogens. Transcriptomics data can be a useful initial screening approach to analyse the native promoter strength. In fact, a transcriptomics analysis study in *M. thermoacetica* showed that transcription of the gene encoding glyceraldehyde-3-phosphate dehydrogenase (G3PD) was high and constitutive during both heterotrophic and autotrophic growth of the organism (Kita *et al*. 2013). The authors further demonstrated that this promoter could be used for driving the expression of non-native genes such as a gene encoding a lactate dehydrogenase (Iwasaki *et al*. 2017) and a thermostable kanamycin resistance gene (Iwasaki *et al*. 2013). This is, to date, the only strong constitutive promoter identified for *M. thermoacetica*. Similarly, a few native constitutive promoters have been identified in other acetogens. The native promoter of the *acsA* gene, P*_acsA_* was used for overexpression of target genes in *C. autoethanogenum* (Liew *et al*. 2017), while expression of genes involved in butyrate production in *C. ljundahlii* were placed under the transcriptional control of the native *pta* gene promoter, P*_pta_* (Ueki *et al*. 2014). Likewise, the strong acetogenic promoter, P*_ack_* from *A. woodii* was used to drive the expression of an acetone-producing operon in *A. woodii*, but it did not increase acetone production as compared to two non-native promoters used: P*_thl_* from *C. acetobutylicum* and P*_pta_* from *C. ljungdahlii* (Hoffmeister *et al*. 2016). The putative strong promoter of the S-layer protein of *T. kivui* also allowed the expression of hydrogen-dependent carbon dioxide reductase genes in complementation experiments (Jain *et al*. 2020). These strategies rely on using one of the host's own promoters for expression of target genes and, to date, only a few non-native constitutive promoters have been used in acetogens. Woolston *et al*. (2018) mediated the expression of guide RNAs for CRISPR interference in *C. ljungdahlii* using an engineered P4 promoter, previously tested in *Clostridium cellulolyticum* (Xu *et al*. 2015), and also used the thiolase promoter, P*_thl_* from *C. acetobutylicum*. As mentioned, this P*_thl_* promoter has also been successfully used to control the expression of acetone-producing genes in *A. woodii* and, in fact, led to higher acetone production than a native promoter (Hoffmeister *et al*. 2016). In addition, after testing several exogenous promoters with the *lacZ* reporter assay, the P*_thl_* promoter was chosen to drive expression of *cas9* in *C. ljungdahlii*, while *C. acetobutylicum* P*_araE_* promoter was responsible for expression of guide RNAs when developing a CRISPR-Cas9 tool for this acetogen (Huang *et al*. 2016). A thiolase promoter from *Clostridium tyrobutyricum* (Yu *et al*. 2011) has been shown to be functional in *C. carboxidivorans* and was harnessed for improved butanol and ethanol production (Cheng *et al*. 2019). In addition, complementation of the *pyrE* gene, under the control of a promoter of the gyrase gene of *Thermoanaerobacter* sp. X514, restored wild-type phenotype in a *pyrE* mutant strain of *T. kivui* (Basen *et al*. 2018). Shin *et al*. (2019) also opted for a strategy similar to Woolston *et al*. (2018) when adapting a CRISPR-Cas9 tool in *E. limosum*. While *cas*9 was controlled by an inducible promoter, the guide RNAs were constitutively expressed under the control of a synthetic *E. coli* promoter, Bba_J23119 (Larson *et al*. 2013). Lastly, Yang *et al*. (2017) created a library of artificial promoters by randomising the regions flanking the -35 and -10 regions of the *C. acetobutylicum* P*_thl_* promoter and further modifying the RBS sequence. Although, initial promoter screening and testing were performed in the non-acetogenic industrial chassis *C. acetobutylicum*, the authors investigated the activity of the engineered promoters in the acetogen, *C. ljungdahlii*, obtaining similar results to *C. acetobutylicum*, further supporting the wide-spread success of clostridial tools in acetogens. The promoters also allowed an increase of product yield, illustrating the relevance of synthetic promoters for target expression in acetogens. These studies have, thus, identified a few native and non-native strong promoters for the expression of target genes in several acetogens. However, strong constitutive expression might be deleterious for some applications leading, for example, to toxicity. Instead, fine-tuned expression via inducible systems might be more suitable, and several inducible promoters have been developed for some acetogens. For example, a lactose-inducible system, first isolated from *C. perfringens* strain 13 (Hartman, Liu and Melville 2011), relies on the transcriptional regulator BgaR, acting on the P*_bgaL_* promoter and allowed high induction level in *C. ljungdahlii*, where it was applied for disruption of ethanol and acetate production (Banerjee *et al*. 2014). However, this promoter remained leaky, with significant transcription taking place in the non-induced state (Banerjee *et al*. 2014; Woolston *et al*. 2018), preventing its use for specific tools such as an inducible CRISPR interference tool (Woolston *et al*. 2018). Poor repression has also been observed in *A. woodii* (Beck *et al*. 2020). On the contrary, tetracycline-inducible promoters have proven to be tightly repressed in the absence of an inducer in several acetogens. For example, the P*_2tetO1_* promoter, first shown to work in *C. acetobutylicum* (Dong *et al*. 2012), offered a tighter repression in *C. ljungdahlii* with lower expression levels compared to P*_bgaL_*, and was applied for CRISPR interference (Woolston *et al*. 2018). Similar results have been observed for *A. woodii* (Beck *et al*. 2020) and *E. limosum* (Shin *et al*. 2019). In addition, a library of tetracycline-inducible promoters, Tet3no variants was constructed to identify the most suitable promoter for the CRISPR-Cas9 tool in *C. autoethanogenum* (Nagaraju *et al*. 2016). For the intended application, the authors chose a variant inducing high expression, but poorly repressed in the absence of an inducer. Two additional promoters, the lactose-inducible P*_fac_* promoter and the theophylline-inducible P*_ack-theo_* promoter, were also tested in *A. woodii* but did not offer tight repression and high induction, respectively (Beck *et al*. 2020). These inducible promoters are useful tools for strain engineering purposes to allow fine-tuned expression that is essential for some applications such as CRISPR-Cas tools. Moreover, to test promoter activity, assays with different reporter genes, including the chloramphenicol acetyltransferase gene, *catP* or the β-glucuronidase gene, *gusA* (Banerjee *et al*. 2014; Nagaraju *et al*. 2016; Woolston *et al*. 2018; Beck *et al*. 2020), can be performed. In addition, fluorescent reporter genes such as the green fluorescent protein (GFP) gene can be an alternative reporter system to those mentioned previously. Initially, these systems required oxygen for the fluorophore to fold properly. However, several fluorescent reporter genes have now been engineered to work under anaerobic conditions. For example, the anaerobic fluorescence marker evoglow^®^ has previously been used in *C. ljungdahlii* (Molitor *et al*. 2016), and the oxygen-independent CreiLOV marker (Mukherjee *et al*. 2015) is also functional in *E. limosum* (Shin *et al*. 2019). The latter fluorescent reporter also has an increased thermostability, suggesting a potential application in thermophilic acetogens. These different reporter systems are crucial to assay the promoter activity and further consolidate the genetic toolbox available for genetic manipulations of acetogens.

##### Ribosome-binding sites

While promoters control transcription, ribosome binding sites (RBS) dictate translational activities; therefore, also impacting gene expression levels. Despite their impact on gene expression, fewer studies tend to focus on RBS, as promoter engineering seems to be the predominant strategy when manipulating genetic parts. Thus, it is not surprising that very few studies have focused on RBS in acetogens. Ueki *et al*. (2014) modified the RBS of the crotonase gene, *crt* in *C. ljungdahlii* by increasing the distance between the RBS and the translation initiation codon. This modification led to the increased expression of Crt, further improving butyrate production. Similarly, RBS optimisation was explored to improve the production of 3-hydroxybutyric acid in *C. ljungdahlii* (Woolston *et al*. 2018). However, the designed optimised RBS did not significantly increase acid production as compared to the original RBS. Furthermore, a genome-wide analysis of *E. limosum* (Song *et al*. 2017) allowed identification of the highly conserved Shine-Dalgarno motif, GGAGR, with a 5-to-10 nucleotide spacer as the consensus RBS for this organism. This study also showed that the genes encoding the enzymes of the WLP, the Rnf (ferredoxin:NAD^+^ oxidoreductase) and ATP synthase complexes, all shared the RBS, AGGAGG. It is also worth noting that computational tools such as RBS Designer (Na and Lee 2010) and RBS Calculator (Salis, Mirsky and Voigt 2009) can help design synthetic RBS for metabolic engineering purposes. For example, the RBS Calculator can create RBS to induce a specific translational rate. The examples presented above are the only studies that implemented or discussed RBS modifications in acetogens. However, these regulatory elements are important to modulate gene expression, especially for metabolic engineering purposes and hence, further work on RBS in acetogens is still needed.

##### RNA-based methods

In addition to using genetic parts-based tools, other genetic engineering strategies can be applied to modulate gene expression levels. Most of these techniques are mediated by synthetic single-stranded RNA molecules that are complementary to the target sequence's mRNA. These methods offer temporary downregulation, as they do not modify the genome. In addition, they also allow the study of essential genes, for which deletion is not viable, by causing a knockdown instead of a knockout. Indeed, binding of these exogenous RNA molecules to the target will either induce degradation of the target mRNA or block translation, inducing gene downregulation (Choi *et al*. 2019). These tools are reversible and can be multiplexed, i.e., targeting several genes in the same experiment, and enable to study essential genes that cannot be fully deleted. Although promising, these tools have not been extensively explored in acetogens. Woolston *et al*. (2018) adapted a CRISPR interference tool for *C. ljungdahlii* to block gene transcription induced by the binding of a nuclease-deficient Cas9 and directed by a specific guide RNA to the target sequence. This approach allowed the redirection of carbon flux for increasing 3-hydroxybutyrate production in an engineered strain. In a similar manner, CRISPR interference targeted five genes involved in the WLP in *E. limosum* to further investigate their importance for autotrophic growth (Shin *et al*. 2019). To date, these two reports are the only RNA-mediated downregulation studies published for acetogens. However, several RNA-based methods have previously been used in clostridia (Cho and Lee 2017), further strengthening the potential of these strategies for genetic engineering.

#### Modification of the host's genome

For genetic engineering purposes, desired modifications such as gene insertion, deletion, or mutation in the genome of an acetogenic host can be performed using directed homologous recombination (HR)-mediated methods, through random insertions using mobile elements, and CRISPR-based tools, as discussed in the following sections.

##### Homologous recombination-mediated methods

To test the activity of exogenous enzymes, plasmid-borne genes can be expressed once introduced in the desired host organism. However, in an industrial context, plasmid-mediated expression is not ideal as it requires plasmid maintenance via selective pressure, often requiring expensive selection markers. Instead, target exogenous genes can be inserted into the host's chromosome, allowing higher strain stability and reduced medium cost. Homologous recombination (HR) has been used as a common mean for site-specific insertion of exogeneous genes into the genome (Heap *et al*. 2012). HR can also be exploited to delete or modify target native genes via an HR cassette. HR-based methods require homology arms flanking the target site and the modification template to insert into the chromosome. Many techniques to select for an HR-mediated template insertion have been developed and are reviewed elsewhere in more details (Minton *et al*. 2016). Indeed, to select for integrated mutants, cassette integration requires to give a selective advantage to the mutants, and different auxotrophies can be used for selection of HR-mediated mutations. In this method, genetic manipulation is performed in an auxotrophic mutant strain, allowing complementation of the auxotrophic marker as the selection pressure for successful integration. As mentioned earlier, *pyrF* and *pyrE* genes, involved in uracil biosynthesis, have been used as auxotrophic markers in *M. thermoacetica* and *T. kivui*, respectively, as well as in many other organisms (Donovan and Kushner 1983; Boeke, LaCroute and Fink 1984; Groom *et al*. 2014). Integration of the HR construct harbouring *pyrF/E* and the desired genetic modifications in a mutant background restores the wild-type phenotype; thereby, allowing selection of transformants while introducing the desired genetic modifications. This system was exploited to insert the lactate dehydrogenase gene from *Thermoanaerobacter pseudethanolicus* into *M. thermoacetica* chromosome (Kita *et al*. 2013; Iwasaki *et al*. 2017), and to delete a phosphofructosekinase (Basen *et al*. 2018) and a gene encoding a hydrogen-dependent carbon dioxide reductase complex (Jain *et al*. 2020) in *T. kivui*. A similar approach led to the deletion of genes encoding an aldehyde:ferredoxin oxidoreductase and an aldehyde/alcohol dehydrogenase in *C. autoethanogenum* (Liew *et al*. 2017), as well as the generation of several mutants of *A. woodii* (Schoelmerich *et al*. 2018; Westphal *et al*. 2018; Wiechmann *et al*. 2020). These methods allowed deletion of large sequences, including the *rnf* operon (Westphal *et al*. 2018) and six genes involved in lactate metabolism (Schoelmerich *et al*. 2018), further illustrating the effectiveness of HR combined with auxotrophic markers for genome editing in acetogens. The use of other auxotrophic markers such as leucine and histidine auxotrophies have been reported in model organisms (Pronk 2002; Monneau *et al*. 2016). Recently, genes encoding vitamin prototrophy for pantothenate and thiamine were introduced into *C. autoethanogenum* and *C. ljungdahlii*, further expanding the repository of auxotrophic markers available for acetogens (Annan *et al*. 2019). However, this method required to determine to which vitamin(s) the hosts were initially auxotrophic. The authors also relied on allele-coupled exchange (ACE) (Heap *et al*. 2012) to select double crossover events required for the HR cassette integration. Since double-crossover events are rarer than single-crossover events, the ACE method is designed to facilitate the selection of double-crossover mutants. When HR occurs during ACE, a plasmid-borne allele combines with a genome-borne allele to create a new selectable allele. For example, a promoter-less *pyrE* is inserted downstream of a constitutive promoter in the genome, therefore activating *pyrE* and allowing selection, as applied in *C. acetobutylicum* (Heap *et al*. 2012) and *C. autoethanogenum* (Liew *et al*. 2017). Thus, HR remains a key tool for gene insertion, deletion, or modification, and recently developed techniques have further simplified its application for the selection of mutants. Although auxotrophic markers may be more suitable in an industrial context, antibiotic resistance is still an important mean for the selection of transformants. Similar to *pyrF/E*-selection/counterselection, an antibiotic resistance gene can be inserted into the genome by HR to select for appropriate cassette insertion. However, several selection markers must be used when building a strain with multiple deletions. This process can be challenging as the number of markers available for a specific host can be limited. Instead, the Cre-Lox method allows marker removal once the desired mutation is obtained; thereby, enabling marker recycling. This tool, adapted from the P1 bacteriophage, includes the Cre-recombinase that only induces recombination at two Lox sites flanking the target selection marker (Sauer 1987). Recombination at the two Lox sites causes removal of the selection marker, which can then be reused to select for other modifications. This method allowed iterative construction of an engineered strain of *C. ljungdahlii* for butyrate production (Ueki *et al*. 2014). Moreover, a recent study implemented a phage serine integrase-mediated tool for *C. ljungdahlii* (Huang *et al*. 2019). The authors showed that the phage attachment/integration systems from *Clostridioides difficile* ΦCD27 and *Streptococcus* ΦC31 phages were functional in *C. ljungdahlii* and compatible with each other, as both systems could be used simultaneously. This application, however, required insertion of the bacterial attachment site into the host's genome to allow the plasmid-borne phage attachment site to bind to the genome, enabling a serine integrase-mediated recombination. The authors also combined this new tool with a CRISPR-Cas9 tool (Huang *et al*. 2016) for rapid screening of colonies, when both aforementioned attachment/integration systems were used simultaneously. As a proof-of-concept step, this tool was harnessed for the integration of *C. acetobutylicum* butyric acid pathway in *C. ljungdahlii* genome (Huang *et al*. 2019), illustrating the importance of this method to insert large genetic constructs in acetogens. Thus, the different HR-mediated methods discussed in this section have also been applied to select transformants harbouring the desired genetic modifications during the HR-based genome editing, a key approach for genetic engineering of acetogens.

##### Mobile elements

In addition to HR-based methods, mobile elements have been adapted for implementing directed mutagenesis in different clostridial species (Heap *et al*. 2010). These tools mostly mediate gene disruption, as insertion of the mobile element interferes with the target gene. ClosTron is such a technology that relies on inserting bacterial group II introns to disrupt genes (Heap *et al*. 2007). Site recognition is mediated by base-pairing between the target DNA and intron RNA, which can be engineered to target desired genes. Intron mobility also requires an intron-encoded protein (IEP), which can be removed once mutations have been achieved; thereby, obtaining stable mutant strains. More recently, ClosTron has been used to disrupt genes in the acetogen, *C. autoethanogenum* (Mock *et al*. 2015; Marcellin *et al*. 2016). Indeed, Marcellin *et al*. (2016) created mutant strains using the ClosTron method for experimental validation of computational simulation results. More specifically, the authors targeted genes involved in gluconeogenesis to investigate the energy requirement of this pathway. Similarly, energy conservation was studied by disrupting genes encoding hydrogenases using ClosTron in another report (Mock *et al*. 2015). This tool was also applied to the acetogen, *C. ljungdahlii* to delete *adhE1*, abolishing ethanol production (Bengelsdorf *et al*. 2016). Thus, the mobile elements-based ClosTron is a powerful tool for implementing genetic modifications in the host genome by making gene disruptions, and has already been adapted for use in some acetogens. However, there are some limitations associated with ClosTron, especially relating to polar mutations which might impact phenotypic traits. In addition, ClosTron application is limited to gene disruption only, as gene insertion cannot be mediated with this tool.

A recent study relied on a transposase-mediated integration method to introduce an acetone pathway into *C. ljungdahlii* genome (Philipps, de Vries and Jennewein 2019). Indeed, after optimisation of the conjugation protocol, the *Himar1* transposase from *Haematobia irritans* (Lampe, Churchill and Robertson 1996), controlled by *Staphylococcus xylosus* xylose-inducible promoter (Sizemore *et al*. 1991), allowed the insertion of an erythromycin cassette into *C. ljungdahlii* genome. The authors further applied this method to integrate a complete metabolic pathway into the genome, leading to acetone production in this host. This method enables insertion of large cassettes into the host's genome, relying solely on the transposase and inverted terminal repeats that are flanking the cassette and acting as recognition sites for the transposase. However, the authors reported that the integration locus was random, which might cause downstream effects. Nonetheless, this technique is promising to easily insert large genetic constructs in acetogens.

##### CRISPR-based tools

The methods described above have been used to genetically manipulate many organisms for years. However, the advent of the genome-editing tool, CRISPR-Cas9 (Clustered Regularly Interspaced Short Palindromic Repeats-CRISPR associated protein 9) has revolutionised the entire genetic modification toolbox available for modifying a host's genome (Barrangou *et al*. 2007; Jinek *et al*. 2012; Cong *et al*. 2013). CRISPR‐Cas systems are widely distributed in many bacteria and archaea, where they act as a natural defence system capable of recognising and cleaving invasive foreign DNAs. These properties, and in particular those of the *Streptococcus pyogenes* CRISPR-Cas9 system, have been exploited and engineered for site-specific editing of target DNA sequences in host genomes. In this method, an engineered single guide RNA molecule guides the introduced endonuclease Cas9 to the target site, allowing site-specific double-strand breaks. In eukaryotic organisms, non-homologous end joining (NHEJ) allows to repair the breaks, creating random mutations when this option is desired. However, in prokaryotes unable to perform NHEJ (Joseph, Kim and Sandoval 2018; Vees, Neuendorf and Pflügl 2020) such as acetogens, genome editing via CRISPR-Cas9 relies on HR-based replacement of the target sequence with the desired mutant allele and subsequent elimination of the wild-type population through RNA-guided Cas9 cleavage of the parental allele. Thus, conventional allelic exchange mechanisms generate mutants, while CRISPR-Cas9 allows selection of mutants harbouring the desired allele from mixed populations and therefore, immune to cleavage, unlike the wild-type cells. More recently, other CRISPR-Cas systems such as CRISPR-Cas12a and endogenous CRISPR-Cas systems have gained interest for genome editing in order to alleviate some of the drawbacks of CRISPR-Cas9, including Cas9 toxicity or off-target cleavage.

CRISPR-Cas9-based tools have been adapted for many organisms, including some acetogens. In the first report of a CRISPR-Cas9 tool in an acetogen, Huang *et al*. (2016) used the *S. pyogenes* Cas9 nuclease for guided targeting of four *C. ljungdahlii* genes, allowing successful mutant selection for all the targets. In this proof-of-concept study, the expression of *cas9* was placed under the strong constitutive promoter, P*_thl_* from *C. acetobutylicum*. A similar approach was later undertaken in *C. autoethanogenum* to knockout the *adh* (alcohol dehydrogenase) and *bdh* (2,3-butanediol dehydrogenase) genes. However, a tetracycline-inducible promoter was used to regulate the expression of *cas9*, as constitutively expressing *cas9* was not viable for this system due to the potential Cas9 toxicity (Nagaraju *et al*. 2016). A similar tetracycline-inducible system has recently allowed the CRISPR-Cas9-mediated deletion of three genes in *E. limosum* (Shin *et al*. 2019). Controlled expression of *cas9* is particularly important for successful CRISPR-Cas9 applications, as nucleases are generally toxic for both the *E. coli* donor and the acetogen target. In fact, it was later shown that the *cas9* gene on the vectors utilised by Huang *et al*. (2016) was predisposed to the acquisition of mutations in the *E. coli* donor, leading to the production of a truncated Cas9 protein (Ingle *et al*. 2019). This truncated Cas9 protein appeared to be a nickase; therefore, only cleaving a single strand of the DNA, which is less toxic than the double strand break induced by a native Cas9 (Li *et al*. 2016). More recently, a novel riboswitch-based editing tool, RiboCas, has been engineered to overcome excessive Cas9 toxicity (Cañadas *et al*. 2019) by tightly repressing *cas9* expression using a theophylline-inducible riboswitch. Originally demonstrated in four non-acetogenic clostridial species, it has now been shown to function effectively for the generation of mutants in *C. autoethanogenum* (Seys *et al*. 2020). This recent study also describes a strategy for ‘gold standard’ complementation, in which a unique 24-nucleotide ‘bookmark’ sequence incorporated into the mutant allele acts as a guide RNA target during its CRISPR-Cas9-mediated replacement with the wildtype allele. These examples show that CRISPR-Cas9 tools are proving to be highly effective for the rapid isolation of marker less mutations in acetogenic hosts. Although CRISPR-Cas9 systems are yet to be exploited in thermophilic acetogens, a thermostable Cas9 has already been engineered and used in thermophilic hosts (Mougiakos *et al*. 2017), further suggesting that a CRISPR-Cas9 tool, although challenging, can be successfully adopted in these hosts.

As mentioned, *cas9* expression is toxic in most host organisms, and its large size further reduces the transformation efficiency of a host. Thus, more recent studies focus on harnessing other CRISPR-Cas-based systems such as CRISPR-Cas12a and endogenous CRISPR-Cas systems. The various CRISPR-Cas systems are all relying on Cas proteins, guided by RNAs, and complementary to the target sequence to induce a DNA nick or double strand break. However, specific Cas proteins are associated with each system, further grouping similar systems into classes and types (Haft *et al*. 2005; Makarova *et al*. 2015; Westra, Buckling and Fineran 2014). In addition, the protospacer adjacent motif (PAM) sequence is specific to each Cas protein and is important to increase the targeting efficiency while preventing hosts’ self-targeting. For example, Cas12a recognises the PAM sequence TTN, a better-suited sequence than the Cas9 PAM, NGG for working in A-T-rich species. In fact, a CRISPR-Cas12a tool, also called CRISPR-Cpf1, has already proven to be useful for genome editing in *Clostridioides difficile* (Hong *et al*. 2018) and *Clostridium beijerinckii* (Zhang *et al*. 2018a), targeting genes important for pathogenesis and ethanol production, respectively. A similar system was recently adapted and implemented in the acetogen *C. ljungdahlii*, allowing the redirection of carbon flux (Zhao *et al*. 2019). This approach required to test several Cas12a enzymes, as this study showed that finding the less toxic variant depends on the choice of host species. After identifying *Francisella tularensis* Cas12a as the best candidate for *C. ljungdahlii*, the *pyrE* gene was initially targeted as a proof-of-concept approach, and the successful target deletion demanded a further optimisation of the electroporation protocol. Tool efficiency was then investigated for three additional genes (Zhao *et al*. 2019).

In addition to the use of inducible promoters and different Cas nucleases, the endogenous variant of CRISPR-Cas systems can be adopted to alleviate the associated toxicity of expressing most exogenous CRISPR-Cas systems in many host organisms. As toxicity remains a key limitation to efficient CRISPR-mediated genome editing, especially in organisms with a low transformation efficiency such as clostridia, several studies in this species (Pyne *et al*. 2016; Zhang *et al*. 2018b) have reported to harness the hosts’ endogenous CRISPR-Cas systems for genome modification purposes (McAllister and Sorg 2019). Indeed, 40% of the sequenced bacteria harbour CRISPR-Cas systems (Grissa, Vergnaud and Pourcel 2007), with CRISPR-Cas9 being one of the least common types. Both aforementioned studies in clostridia first identified the putative PAM sequences through the computational analysis, and later tested experimentally which ones were recognised by the hosts’ Type I-B CRISPR system. Pyne *et al*. (2016) successfully deleted the *cpaAIR* gene in *Clostridium pasteurianum* with the endogenous CRISPR-Cas system by expressing a synthetic CRISPR array, harbouring the guide RNA for successful targeting of *cpaAIR*. The authors also showed that this approach yielded a better editing efficiency than an exogenous CRISPR-Cas9 tool. A similar approach was also adopted for deletion of several targets in *Clostridium tyrobutyricum* (Zhang *et al*. 2018b). However, in this case, the native lead promoter sequence of the CRISPR array could not be used to drive expression of the synthetic CRISPR array, potentially due to toxicity; instead, a lactose-inducible promoter was proved suitable. After investigating the impact of spacer length on editing efficiency, Zhang *et al*. (2018b) multiplexed this tool and deleted *spoA* and *pyrE* simultaneously by adding the two required spacers on the same synthetic CRISPR array. The authors further applied this tool for improved butanol production in *C. tyrobutyricum*. Although experimental exploitation of endogenous CRISPR-Cas systems has not been explored in acetogens yet, this approach seems promising for organisms with low transformation efficiencies as exemplified in the two clostridial species mentioned above. Unfortunately, despite yielding better editing efficiencies than CRISPR-Cas9 tools in these studies, Zhang *et al*. (2018b) still reported some level of toxicity when the CRISPR array was strongly expressed and observed off-target editing depending on the spacer length. In addition, although many bacterial species carry endogenous CRISPR-Cas systems, some do not natively harbour these proteins such as the acetogen *C. ljungdahlii* (Pyne *et al*. 2016), preventing implementation of this method. Nonetheless, given the challenges of transforming acetogens, editing tools based on endogenous CRISPR-Cas systems have the potential to stand out as a key approach for modifying these hosts.

Lastly, other derivatives of CRISPR-Cas systems such as CRISPRi (CRISPR interference) have further expanded the genetic toolbox available for some acetogens. For instance, CRISPRi was successfully mediated with a deactivated or dead Cas12a for several targets in the acetogen, *C. ljungdahlii* (Zhao *et al*. 2019). The authors elegantly showed how target sites within the translation initiation region impacted interference efficiency and applied their findings to manipulate the host's carbon flux. The use of a dead Cas9 (dCas9) to knockdown gene expression through RNA interference was also described for the downregulation of *pta* and *aor2* genes in *C. ljungdahlii* (Woolston *et al*. 2018), and of *fhs1*, *folD*, *acsC*, *acsD*, and *ptsF* genes in *E. limosum* (Shin *et al*. 2019). This approach is particularly useful when the target genes are essential, as it still allows gene downregulation. Moreover, dCas9 can be combined with other enzymes to create base-editing tools. For example, Xia *et al*. (2020) fused *S. pyogenes* dCas9 with *Petromyzon marinus* cytidine deaminase (Banno *et al*. 2018), which then allowed the site-directed substitution of cytosine to thymine. The authors first targeted the *pta* gene in *C. ljungdahlii* to assess the efficiency of this tool and showed that it modified bases predominantly in a hot-spot editing window. Following a computational analysis of genome-wide potential target sites, four genes (*adhE1*, *adhE2*, *aor1* and *aor2*) were edited with this tool, creating a premature STOP codon. The obtained strains had similar fermentation profiles to mutant strains harbouring target deletions, further promoting this tool as a key method for gene disruption. Although the reported base-editing tool was highly efficient in *C. ljungdahlii*, two limitations were noted: off-target editing led to downstream phenotypic impacts and a low frequency of the Cas9 PAM sequence (NGG), essential for efficient targeting in A-T-rich acetogens, limited the potential target sites.

## METABOLIC ENGINEERING CHALLENGES RELEVANT TO ACETOGENS

The availability of robust and reliable genetic tools not only help overcome the challenges of successful DNA transfer and required genetic modifications in a host, but also allow further metabolic engineering efforts in it to improve its performance. Metabolic engineering primarily involves the application of many of the discussed genetic engineering and other specialised tools to enable the optimised production of native and non-native compounds, or the diversion of metabolic fluxes to increase the product yield and titre in an engineered chassis. Such improvements are essential for a wider use of the chosen organism in an industrial context to not only broaden its scope and applications but also improve its robustness and productivity. Thus, some of the genetic tools described above have been applied for strain engineering purposes in different acetogens, leading mostly to the production of non-native compounds in these hosts. These achievements, further detailed below, are crucial to enable a broader and diverse industrial use of these organisms. While metabolic engineering mainly tends to focus on producing specific targets, many other aspects are important for this diverse discipline, and a variety of strategies have been reported to increase product titres or diversify fermentation profiles of engineered chassis. As acetogens have only been recently made genetically accessible, the metabolic engineering strategies explored for these organisms remain limited. The following sections will cover only the strategies recently attempted in acetogens, albeit limited. Other publications (Liew *et al*. 2016; Chae *et al*. 2017; Humphreys and Minton 2018; Choi *et al*. 2019) have covered similar and additional strategies for acetogens and other organisms.

### Production of non-native compounds in acetogens

As mentioned, acetogens are industrially important chassis organisms due to their metabolic diversity and versatility in substrate use. Indeed, they could be used as key cell factories for the production of target chemicals from C1-gases. As such, the genetic tools described above have been applied to achieve different metabolic engineering purposes in these hosts (Table 2). For example, several non-native compounds have been successfully produced in acetogens. Both *C. ljungdahlii* and *A. woodii* have been engineered to produce acetone by introducing the acetone biosynthetic pathway from *C. acetobutylicum* (Banerjee *et al*. 2014; Hoffmeister *et al*. 2016). A poly(3-hydroxybutyrate) pathway has recently been introduced in the understudied acetogen, *Clostridium coskatii* (Flüchter *et al*. 2019). Other non-native compounds produced in acetogens include butanol (Ueki *et al*. 2014), isoprene, and mevalonate (Diner *et al*. 2018). Notably, the application of available genetic toolkits for acetogens has also allowed manipulation of native metabolic features for improved performance of the hosts. For example, deletion of the lactate dehydrogenase gene (*ldhA*) in *C. autoethanogenum* increased the ethanol production in this chassis (Nagaraju *et al*. 2016). The metabolic performance of *C. carboxidivorans* has also been improved by expressing *C. acetobutylicum* genes to increase both ethanol and butanol production (Cheng *et al*. 2019). Several other reports have mentioned the successful engineering of acetogens for a desired function as summarised in Table 2. However, reports of metabolic engineering efforts in acetogens remain scarce. In addition, more complex metabolic engineering endeavours such as the introduction of fully synthetic pathways or multi-layered genetic control circuits have yet to be adopted in acetogens. It is also worth noting that, although most genetic applications described here aim at manipulating the host's metabolism, genetic tools are also important for fundamental research and improving our understanding of the host's metabolic features. For example, deletion of the *rnf* operon in *A. woodii* inhibited autotrophic growth, further linking the Rnf complex to energy conservation (Westphal *et al*. 2018). Similarly, lactate metabolism was investigated in a mutant *A. woodii* strain and was shown to require the *lctBCDEF* operon for lactate catabolism (Schoelmerich *et al*. 2018). Therefore, genetic tools are crucial to create mutant strains for both fundamental studies and industrial applications.

### Computational pathway design and analysis

Ideally, most building blocks for industrially important chemicals can be biologically produced to create a more sustainable chemical industry. As mentioned, microbial hosts such as acetogens have already been metabolically engineered through the insertion of natural biosynthetic pathways to produce industrially important chemicals, including acetone, ethanol, butanol, isoprene, and mevalonate. Although this strategy expands the number of compounds produced by these hosts, it still remains limited as few relevant biosynthetic pathways can be found in nature. Instead, fully synthetic pathways can be created and engineered to further benefit from the fermentation abilities of these host organisms. In fact, several recent computational tools, reviewed elsewhere in more details (Long, Ong and Reed 2015; Wang *et al*. 2017; Ko *et al*. 2020), have been developed to guide the design of metabolic engineering strategies and are especially useful to test the feasibility of designed pathways for their experimental implementation in suitable hosts. These tools often rely on the host's genome-scale metabolic model (GEM), *i.e*., a mathematical reconstruction of the host's metabolic network (Santos, Boele and Teusink 2011; Gu *et al*. 2019). The reconstruction compiles gene annotations from genome sequences, data from the literature and biochemical databases, and can be performed using different computational tools such as COBRA (Heirendt *et al*. 2019), RAVEN (Wang *et al*. 2018), and the Model SEED (Henry *et al*. 2010). GEMs have been constructed for a wide range of organisms, including the acetogens *C. ljungdahlii* (Nagarajan *et al*. 2013), *M. thermoacetica* (Islam *et al*. 2015), and *C. autoethanogenum* (Norman *et al*. 2019), and can be combined with other computational tools (Lewis, Nagarajan and Palsson 2012) to identify different metabolic engineering strategies. For example, the OptKnock (Burgard, Pharkya and Maranas 2003) and RobustKnock (Tepper and Shlomi 2009) algorithms allow the identification of gene deletion targets to improve the host's metabolic performance. The OptKnock algorithm has previously identified genes to be deleted to increase the yield of native and non-native products in *C. ljungdahlii* (Chen and Henson 2016) although selecting the best deletion strategies from the OptKnock analysis was challenging. In another study, a GEM-based analysis in *C. ljungdahlii* showed that depending on the gas composition, the acetate kinase can be essential due to ATP requirements (Nagarajan *et al*. 2013). In addition, integration of omics data with GEMs offers a more systemic approach than GEMs only but is often lacking in model simulations. Recently, omics data was integrated into a GEM of *C. autoethanogenum* (Marcellin *et al*. 2016). This study included experimental data from transcriptomics, proteomics, and metabolomics experiments into the GEM to improve our understanding of this organism's metabolism. Specifically, the authors showed that the Rnf complex was differently regulated under autotrophic and heterotrophic growth conditions, further demonstrating the importance of including omics data in computational analyses. In addition, some computational tools have been designed for exploring other aspects of metabolic engineering such as enzyme engineering (Davey and Chica 2012; Chae *et al*. 2017), but not implemented to any acetogens yet.

### Cell-free systems

Computational tools such as GEMs are useful for preliminary analysis and estimation of pathway yield and feasibility. However, they can overestimate yield or misjudge pathway feasibility, partly due to the lack of experimental data integration in most GEMs. As described earlier, genetic tools for non-model organisms such as acetogens remain limited; therefore, complicating the *in vivo* pathway optimisation with a design-build-test approach. Cell-free systems, thus, can be useful to test chosen pathways or genetic parts for a specific organism. Initially, cell-free systems relied on purified enzymes, which require enzyme overexpression and purification, a difficult step to achieve for some organisms. More recently, crude cell extracts have gained interests, as they mimic the native host cell metabolism better (Karim and Jewett 2016). Indeed, native enzymes remain functional in the cellular extracts to allow cell-free gene expression, as the host's transcription and translation machinery remains intact in the extracts. Exogenous DNA can be added to the cell-free reactions, enabling genetic part testing or pathway prototyping (Silverman, Karim and Jewett 2020). Other applications, such as studying protein complexes or protein modifications, have also been reported and reviewed in more details elsewhere (Silverman, Karim and Jewett 2020). Similar to many new techniques, most applications have been performed and optimised in model organisms such as *E. coli*. For example, pathways for 1,4-butanediol (Wu *et al*. 2017) or limonene (Dudley, Nash and Jewett 2019) have been tested with *E. coli* lysates and further implemented *in vivo*. As cell-free systems are powerful tools for pathway prototyping, recent efforts have been focused on adapting this method to non-model organisms (Yim *et al*. 2019) such as the acetogen, *C. autoethanogenum* (Krüger *et al*. 2020). These publications clearly show that optimisation of the cell-free reaction conditions is species-specific. For example, Krüger *et al*. (2020) demonstrated that higher concentrations of magnesium allowed higher protein yields, and further optimised other reaction parameters, including temperature, amino acid, and DNA concentrations, specifically for *C. autoethanogenum*. Then, the authors used the optimised cell-free reaction system for genetic part analysis, and tested three native promoters for the expression of a reporter protein, evaluating the promoter strength in a more rapid manner than traditional approaches. Moreover, as a proof-of-concept application, three recombinant enzymes were expressed in the optimised cell-free system of *C. autoethanogenum* at high yields. Another recent study reported a cell-free system for pathway prototyping with *C. autoethanogenum* (Karim *et al*. 2020). The authors optimised pathways for 3-hydroxybutyrate (3-HB) and *n*-butanol production by investigating different enzyme combinations and ratios. This approach, first applied to 3-HB pathways, allowed identification of the best candidate pathways before implementing them in *C. autoethanogenum*. In addition, to reflect enzyme ratios, the study aimed at correlating enzyme concentrations in the cell-free systems and promoter strength *in vivo*. A similar method was then successfully applied to optimise the production of *n-*butanol, which required implementation of a much longer pathway than 3-HB. Karim *et al*. (2020), in accordance with previous cell-free studies on other organisms, showed that there is a strong correlation between cell-free and *in vivo* results for pathway optimisation. Indeed, a *C. autoethanogenum* strain harbouring the best 3-HB candidate pathway identified in the cell-free reactions produced up to 15 g/L of 3-HB in continuous fermentation, a much higher titre than previously reported in any organism. Therefore, cell-free systems stand out as a key tool for pathway analysis and optimisation, especially for non-model organisms with limited genetic tools. Although only these limited number of studies have been reported for acetogens to-date, these recent results, highly transferable between *in vitro* and *in vivo* systems, promote cell-free systems as an important new technique for metabolic engineering efforts in acetogens.

### Codon optimisation and harmonisation

The genetic code is redundant as one amino acid can be encoded by multiple codons. However, a species-specific codon usage bias exists. Indeed, a preferred codon is predominantly used over other codons for the same amino acid, and the identity of this frequently used codon varies between species (Webster, Teh and Ma 2017). This bias has been linked to tRNA concentrations, as rare codons are associated with scarce tRNAs (Welch *et al*. 2009). Upon identifying the codon usage bias, this species-specific parameter is considered when expressing heterologous proteins in the concept of codon optimisation. This method relies on changing the DNA sequence of the target protein by substituting the rare codons for frequently used codons in the expression strain, as silent synonymous mutations are thought to have minimal impacts. Many algorithms have been designed for codon optimisation, most of which are in-house algorithms from DNA synthesis companies. Publicly available algorithms include Eugene (Gaspar *et al*. 2012), COOL (Chin, Chung and Lee 2014), and CodonWizard (Rehbein *et al*. 2019). Several studies have shown that codon optimisation of heterologous proteins successfully led to increased product yields in bacterial hosts (Menzella 2011; Šnajder *et al*. 2015; Wang *et al*. 2015), mainly due to a higher and faster protein translation. Similarly, Krüger *et al*. (2020) found that luciferase yield was higher in cell-free reactions when the gene sequence was codon-optimised for *C. autoethanogenum*. However, the increased translational speed caused by codon optimisation has been shown to be detrimental in some studies, leading to insoluble proteins and formation of inclusion bodies (Angov *et al*. 2008). It was hypothesised that rare codons allow ribosomal pausing, as it was observed that rare codons are mostly found in domain boundaries while preferred codons are associated with structural domains such as α-helices (Shabalina, Spiridonov and Kashina 2013). These findings suggested that the occurrence of rare codons in these domains is important for ribosomal pausing, allowing partial protein folding. To apply this hypothesis to heterologous protein expression, Angov *et al*. (2008) created a codon harmonisation algorithm, allowing to maintain rare codons within the DNA sequence but adapting the sequence to the expression strain's codon usage bias. The authors further showed that codon harmonised sequences induced a higher level of protein expression. Codon harmonisation has led to increased protein and product yields in other studies (Kulmala, Huovinen and Lamminmäki 2017; Punde *et al*. 2019). Therefore, codon usage bias clearly plays an important role in the expression of heterologous proteins although codon optimisation and harmonisation remain poorly understood. In fact, Claassens *et al*. (2017) compared codon optimisation and harmonisation for six target proteins in *E. coli* and found that the two strategies impacted the proteins differently, suggesting that some constraints were intrinsic to the protein itself rather than the expression strain. It was also noted that mitigating the codon usage bias does not benefit all proteins, especially when the native strain and the expression strain are phylogenetically close (Mignon *et al*. 2018). This is further illustrated by the successful expression of *C. acetobutylicum* acetone and butyrate producing genes in *A. woodii* (Hoffmeister *et al*. 2016) and *C. ljungdahlii* (Ueki *et al*. 2014), respectively, without codon optimisation or harmonisation. In addition, amino acid starvation directly impacts charged tRNA concentrations, therefore complicating codon usage and preventing reliable prediction (Welch *et al*. 2009). Although codon usage bias and its impacts on heterologous protein expression have not been extensively considered for work in acetogens, this topic is relevant for metabolic engineering and has been shown to enhance protein expression and product yield in other microbial hosts.

### Acetogenic energy limitations

The implementation of most metabolic engineering strategies is not straight-forward, even in model organisms. This aspect, perhaps, illustrates the gaps in our understanding of hosts’ metabolism. Metabolic engineering efforts, especially in acetogens, are further complicated by the energy limitations specific to these host organisms. Indeed, during the conversion of C1-gases into fermentation products by the WLP, one ATP molecule is required for the methyl branch, while the conversion of acetyl-CoA to acetate creates one ATP molecule; therefore, yielding no net ATP in the process (Drake, Gößner and Daniel 2008; Ragsdale and Pierce 2008). However, this ATP balance is essential during the autotrophic growth of acetogens. In fact, it has previously been attempted to delete the two putative phosphotransacetylase genes responsible for the formation of acetate from acetyl-CoA in *M. thermoacetica* by replacing them with a gene encoding lactate dehydrogenase from *Thermoanaerobacter pseudethanolicus* (Iwasaki *et al*. 2017). Although this allowed the production of lactate under heterotrophic conditions, autotrophic growth of the mutants was not viable as the deletions prevented ATP formation via the WLP. However, alternative routes for the ATP synthesis such as the arginine deiminase pathway have been proposed in acetogens. In fact, arginine boosts the *C. autoethanogenum* growth under both heterotrophic and autotrophic conditions as it allows ATP generation (Valgepea *et al*. 2017a). The importance of the arginine deiminase pathway is further supported by recent results, where *C. autoethanogenum* arginine deiminase pathway was implemented in *A. woodii*, leading to an increased ATP production (Beck *et al*. 2020). Furthermore, as there is no net ATP gain via the autotrophic WLP, acetogens have evolved different energy-conserving mechanisms to replenish pools of cofactors and generate ATP, reviewed in details elsewhere (Bertsch and Müller 2015; Schuchmann and Müller 2016). Indeed, ATP availability dictates metabolic engineering in acetogens and must be taken into account when designing engineering strategies.

## SUMMARY AND FUTURE OUTLOOK

Acetogens present several environmental benefits for sustainable production of fuels and chemicals. Their WLP allows the conversion of direct and indirect greenhouse gases, CO_2_ and CO, into different products during the autotrophic gas fermentation; thereby, reducing environmental carbon footprint. This aspect is particularly important and attractive for the implementation of industrial-scale gas fermentation processes to enable biological synthesis of products from C1-gases. However, potential industrial applications will involve strain engineering to improve performance and cost-effectiveness of the envisioned process. Such refinements will be reliant on the exploitation of synthetic biology and metabolic engineering approaches. Some progress in this respect has been made in recent years with a number of acetogen chassis. Implementation of such strategies absolutely relies on reliable and efficient DNA transfer processes. However, to achieve this, several challenges, including stable plasmid replication or bacterial native restriction-modification barriers, must first be overcome. Once DNA transfer has been established, different genetic elements such as promoters or RBS can then be engineered, primarily to manipulate gene expression, a crucial requirement for implementing metabolic engineering strategies. In addition, several methods to achieve genetic modifications, most of which have already been adapted for other acetogens, can then be explored for metabolic engineering purposes. Metabolic engineering efforts in acetogens are, however, constrained by their energy requirements, more specifically, the ATP availability during gas fermentation. However, several computational tools are currently available to guide strategy design and predict modification outcomes. Ideally, omics data would need to be included in these computational analyses to further constrain results. It is also worth mentioning that metabolic engineering in acetogens is currently in its early stages but some challenges relevant to metabolic engineering in general, such as pathway toxicity or regulatory circuits, will need to be addressed in the future. Lastly, gas fermentation at a large scale can be a challenging undertaking because various parameters such as gas solubility and purity can affect its outcome. Thus, constant efforts focusing on creating and improving genetic tools for acetogens to study and manipulate their metabolism are required. While tremendous progress has already been achieved in this area, further work to make most acetogens genetically accessible will have drastic impacts for industrial implications; eventually, contributing to overcoming our dependency on the petrochemical industry.
